# Accuracy of standard bipolar amplitude voltage thresholds to identify late potential channels in ventricular tachycardia ablation

**DOI:** 10.1007/s10840-022-01148-6

**Published:** 2022-02-23

**Authors:** Ivo Roca-Luque, Fatima Zaraket, Paz Garre, Paula Sanchez-Somonte, Levio Quinto, Roger Borras, Eduard Guasch, Elena Arbelo, José Maria Tolosana, Josep Brugada, Lluís Mont

**Affiliations:** 1 Department of Cardiology, Cardiovascular Clinical Institute, Arrythmia Unit, Hospital Clìnic, Universitat de Barcelona. C/Villarroel 170, 08036 Barcelona, Catalonia Spain; 2 Institut d’Investigacions Biomèdiques August Pi I Sunyer (IDIBAPS), Barcelona, Catalonia Spain; 3 Centro de Investigación Biomédica en Red de Enfermedades Cardiovasculares (CIBERCV), Madrid, Spain

**Keywords:** Ventricular tachycardia ablation, Voltage mapping, Scar thresholds, Electroanatomical mapping

## Abstract

**Background:**

Ventricular tachycardia (VT) is caused by the presence of a slow conduction channel (CC) of border zone (BZ) tissue inside the scar-core tissue. Electroanatomic mapping can depict this tissue by voltage mapping. Areas of slow conduction can be detected as late potentials (LPs) and their abolition is the most accepted ablation endpoint. In the current guidelines, bipolar voltage thresholds for BZ and core scar are 1.5 and 0.5 mV respectively. The performance of these values is controversial. The aim of the study is to analyze the diagnostic yield of current amplitude thresholds in voltage map to define VT substrate in terms of CCs of LPs. Predictors of usefulness of current thresholds will be analyzed.

**Methods:**

All patients with structural heart disease who underwent VT ablation in Hospital Clinic in 2016–2017 were included. Maps with delineation of CCs based on LPs were created with contact force sensor catheter. Thresholds were adjusted for every patient based on CCs. Diagnostic yield and predictors of performance of conventional thresholds were analyzed.

**Results:**

During study period, 57 consecutive patients were included (age: 60.4 ± 8.5; 50.2% ischemic cardiomyopathy, LVEF 39.8 ± 13.5%). Cutoff voltages that better identified the scar and BZ according to the LP channels were 0.32 (0.02–2 mV) and 1.84 (0.3–6 mV) respectively. Current voltage thresholds identified correctly core and BZ in 87.7% and 42.1% of the patients respectively. Accuracy was worse in non-ischemic cardiomyopathy (NICM) especially for BZ (28.6% vs 55.2%, *p* = 0.042).

**Conclusions:**

Accuracy of standard voltage thresholds for scar and BZ is poor in terms of LPs detection. Diagnostic yield is worse in NICM patients specially for border zone.

## Introduction

Substrate-based radiofrequency catheter ablation has become a standard procedure for the treatment of scar-related ventricular tachycardia (VT). In recent years, several studies have suggested that complete elimination of the VT substrate results in non-inducibility of tachycardia at the end of the procedure and in fewer VT episodes in the long term [1, 2]. The main mechanism behind scar-related VT is the re-entry. This re-entrant circuit is caused by the presence of a slow conductive channel composed of border zone (BZ) tissue between the scar-core tissue that connects to the healthy tissue, leading to re-entry. These regions are also called conducting channels (CCs). These CCs can be accurately identified with the help of electroanatomical maps (EAMs) obtained during ablation [3]. Initially, to detect this abnormal tissue, only voltage maps were used. In this sense, it is generally accepted that the ventricular bipolar electrograms of healthy myocardium have peak-to-peak amplitudes of > 1.5 mV, while the electrograms in the scar areas demonstrate lower amplitudes (< 0.5 mV) and abnormal fragmentation [4]. These thresholds have been validated mainly for ischemic heart disease in a small cohort of patients [5] and adopted in patients with non-ischemic heart disease also validated in a small cohort of patients [6]. These thresholds were also validated in a porcine infarct model to identify only transmural scars [7]. In addition, these thresholds have been obtained by conventional bipolar mapping catheters lacking contact sensors in the tip. Muñoz et al. compared the scarred areas obtained by traditional voltage mapping with those obtained using a map that included contact force information in 11 patients with VT, demonstrating how this information could improve the traditional map, correcting the points acquired in areas with no contact [8]. Moreover, following the publication of these original validation studies, new high-density mapping catheters have been released, and it has been clearly demonstrated that the accuracy of these catheters is higher than that of conventional bipolar catheters [9, 10]. Finally, the current VT ablation strategy takes not only into consideration the voltage amplitude but also, more importantly, the electrogram (EGM) characteristics, such as fragmentation, late potentials during sinus rhythm or during ventricular pacing, and CCs are more often defined based on these EGM characteristics and pattern activation than on voltage amplification. Indeed, the most accepted endpoint to evaluate the acute success of ablation is the elimination of LPs.

In this study, we aim to investigate the performance of current voltage thresholds for scar and border zones to detect conduction channels determined by EGM characteristics and to determine new voltage thresholds.

## Methods

### Population

We performed a retrospective, cross-sectional, single-center study. Consecutive patients who underwent substrate-based ablation at the hospital clinic from January 2017 to January 2018 were included. Patients for whom ablation was MRI guided were excluded due to insufficient point densities in the EAM.

Structural heart disease was confirmed by cardiac ultrasound and CMR in some patients. Ischemic heart disease was identified by coronary angiogram or CT scan.

The study was carried out according to the Declaration of Helsinki guidelines and the deontological code of our institution. The study protocol was approved by the Ethical Committee of the hospital, and all patients provided informed consent.

### Electrophysiological study and catheter ablation

The procedure was performed under conscious sedation or general anesthesia when epicardial access was anticipated or according to the patient characteristics. The Carto 3 (Biosense Webster, USA) navigation system was used to guide the ablation. Electroanatomical mapping of the LV endocardium was performed for all patients via a transeptal and/or retrograde aortic approach during RV-paced rhythm. Epicardial mapping and ablation were performed via subxiphoid puncture when preprocedural LGE-CMR showed an epicardial scar, endocardial mapping did not reveal an endocardial substrate, electrocardiogram of clinical or induced VT suggested an epicardial origin, or endocardial ablation was unsuccessful. Mapping was performed with a 3.5 mm contact force and magnetic sensing cooled-type ablation catheter (Smart Touch, Biosense Webster, USA) in all patients and additionally a/or a multielectrode catheter (Pentaray; Biosense Webster, USA) for 10% of the patients. A high-density substrate EAM was obtained in each case to identify the presence of CCs. A minimum contact force of 4 g was used for mapping for avoiding overdetection of low voltage areas.

**Fig. 1:**
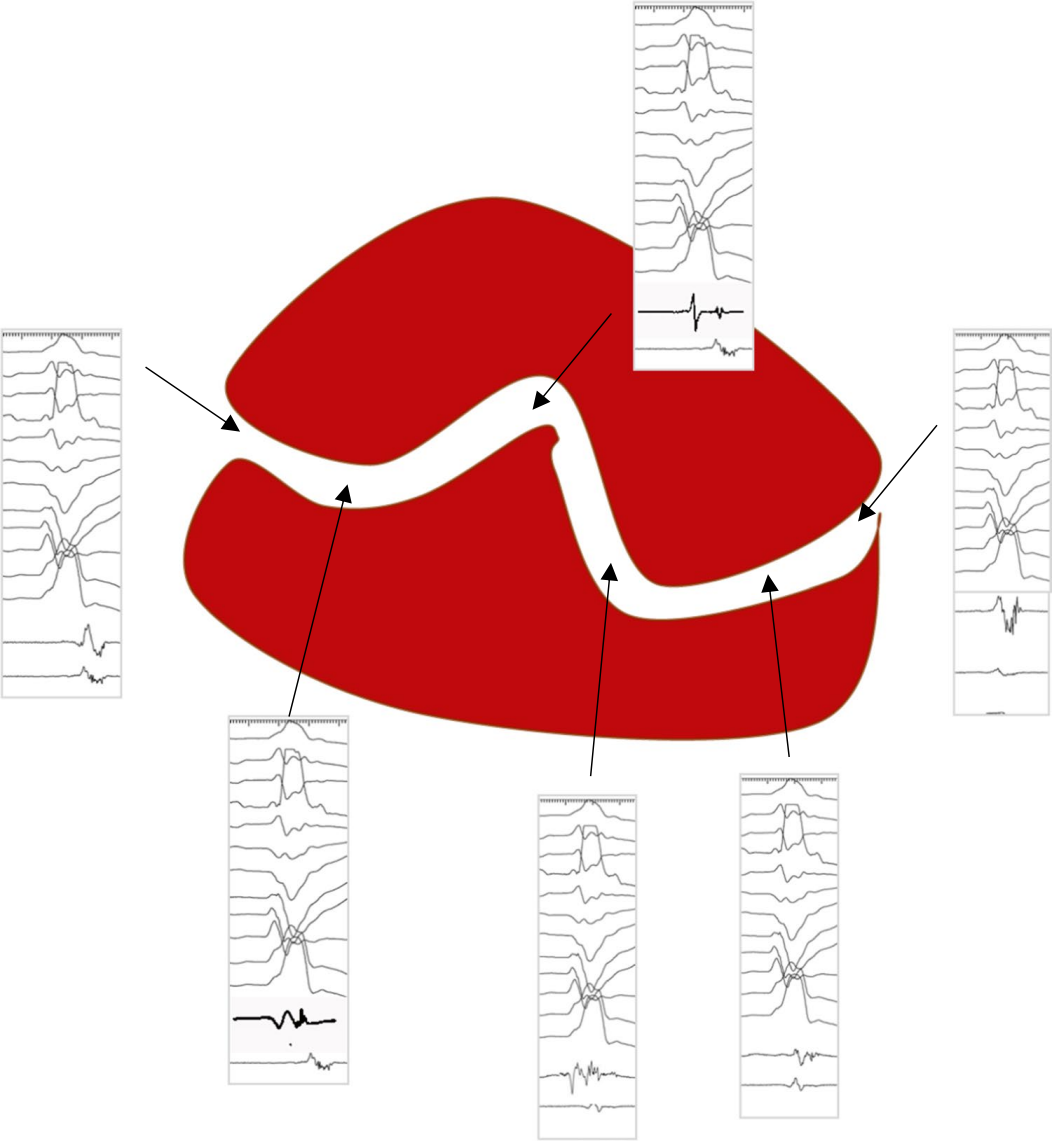
Schematic illustration of conducting channel (CC). A scar in red is drawn with an area of excitable tissue inside the scar. Examples of late potentials with a pattern of activation (more delayed late potentials in the middle of the CC and earlier late potentials in the entrances of the CC) are shown

Bipolar electrograms (EGMs) were recorded with bandpass filters of 10/400 Hz and displayed at a 200 mm/s sweep speed. Bipolar EGMs were classified according to conventional criteria (9.10) as normal (≤ 3 sharp deflections from baseline, amplitude ≥ 1.5 mV, and duration < 70 ms), fractionated (multiple deflections, amplitude ≤ 0.5 mV, and duration ≥ 133 ms), and late (any EGM lasting beyond the end of the surface QRS complex). Fractionated and late EGMs were considered EGMs with delayed components (EGM-DC). Additionally, EGMs with > 1 deflection not meeting the criteria for a fractionated EGM (duration < 133 ms) identified within the scar area or in the periphery of the scar area were considered potential hidden slow conduction EGM (HSC-EGM), as they could represent an EGM-DC hidden by the far-field signal. A closely coupled double or triple extrastimulus was delivered from the RV apex: if the local potential was delayed (with a minimum delay of 10 ms), the response was considered positive, and it was annotated as an HSC-EGM. The bipolar signals were filtered from 10 to 400 Hz and were displayed at 100 mm/sec speeds on the electroanatomical mapping system (CARTO 3, Biosense, Inc.) system. The peak-to-peak signal amplitude of the bipolar electrogram was measured automatically. In case of multicomponent signals, we have analyzed the largest local, that is, the largest late potential by manual change of EAM window of interest if needed to avoid false higher voltage of far-field EGM. Electrograms with delayed components were tagged as such. Our gold standard for defining a CC is the presence of LAVAs and a pattern of activation with late potentials in the middle of the channel and earlier late potentials in the CC entrances (Fig. 1). Subsequently, radiofrequency ablation was started following a previously described 4-step scar dechanneling technique. In brief, all CC entrances were tagged in the EAM. The entrances were defined by the CC electrogram with the shortest delay between the far-field component of healthy or BZ muscle and the local component corresponding to the local activation of myocardial fibers in the scar (usually EGM-DC). Ablation lesions were delivered first at the entrance of the conducting channels. After abolition of the CC entrances (usually EGM-DC and not LPs), the channels were remapped. In case of persistence of LPs inside the CC despite entrance ablation, focal ablation was performed in those areas. Radiofrequency ablation was performed using temperature control (45 °C/40–50 W). The procedural endpoint was both abolition of the CC entrances and LPs and no VT inducibility at the end of the procedure. Voltage mapping was not modified to guide the ablation as, according to scar dechanneling technique, ablation is guided by EGM characteristics as described above.

During the ablation procedure, scar areas were identified using standard voltage cutoff values for dense scars (< 0.5 mV) and BZ scars (< 1.5 mV). However, ablation was based on CC definition rather than on voltage channels as described previously. Postprocedural analysis was performed by an experienced electrophysiologist blinded to the ablation lesions. Manual adjustment of border zone and core scar thresholds was performed to better delineate a CC (defined previously by the presence of LAVAs and a pattern of activation with late potentials in the middle of the channel and earlier late potentials in the CC entrances). The upper and lower voltage that better defined the CC (based on EGM) in terms of corridor of border zone tissue inside a scar area were recorded as the voltage threshold for every patient. These values are considered the best cutoff for the main goal of the study. In case there was no obviously better cutoff, the case was revised by a second experienced electrophysiologist.

### Statistical analysis

Continuous variables are expressed as the mean ± SD and range. Categorical variables are represented by frequencies and percentages. A descriptive analysis of clinical, echocardiographic, and procedure-related variables was performed. For the univariate analysis, the chi-square test or Fisher’s exact test was used for dichotomous categorical variables (including calculation of the 95% confidence interval [CI]), ANOVA was used for non-dichotomous categorical variables, and Student’s *t*-test was used for continuous variables. Candidate variables with *p* < 0.1 in the univariate analyses were incorporated into multivariate stepwise regression models. Two-tailed *p* values < 0.05 were considered statistically significant. Analyses were performed with SPSS software Mac OS version 26.0 (IBM SPSS Statistics, Chicago, USA). The ethics review board of our institution approved this study.

## Results

### Study population

Out of 126 patients who underwent VT ablation during the study period, patients with low-density mapping due to CMR-based ablation and patients without structural heart disease were excluded. Finally, the total sample was 57 patients (87.7% male, age: 60.4 ± 8.5). The most frequent substrates were ischemic heart disease (29, 50.9%) and arrhythmogenic cardiomyopathy (14, 24.6%). The mean left ventricular ejection fraction (LVEF) was 39.8 ± 13.5%. Chronic kidney disease was present in 24.5% of patients, and 21.1% had permanent atrial fibrillation.

The main indication for VT ablation was ICD therapy in 71% of patients (ICD shock in 34%, arrhythmic storm in 15%). In the remaining patients, the indication for ablation included monomorphic VT episodes outside the therapeutic ICD detection window or in patients without ICD.

Cardiac MRI and cardiac CT were performed in 21 (36.8%) and 36 (63.2%) patients, respectively.

The basal characteristics of the study population are shown in Table 1.

**Table 1:** Demographic and clinical baseline characteristics of the population

	Total patients = 57
Age, years	64.67 ± 15.06
Male	51 (90)
Hypertension	22 (36)
Diabetes mellitus	10 (16)
Dyslipidemia	25 (44)
Smoker	31 (54)
Chronic obstructive pulmonary disease	15 (26)
Chronic kidney disease	9 (15)
Permanent atrial fibrillation	21 (37)
Left ventricle ejection fraction (%)	39.77 ± 11.02
Left ventricle end-diastolic diameter (mm)	56.28 ± 13.53
Left ventricle end-systolic diameter (mm)	40.59 ± 13. 98
Arrhythmic storm	8 (14)
Incessant ventricular tachycardia	7 (11)
NYHA functional class	
I	15 (26)
II	8 (14)
III	4 (7)
Ischemic cardiomyopathy	30 (52)
Myocardial infarction localization	
Septal	4 (7)
Antero-lateral	13 (23)
Inferior	12 (21)
Apical	1 (2)
> 2 antiarrhythmic drugs	13 (22)
Area scar	36.67 ± 25.34
Area LV	158.87 ± 43.63

Values are mean ± SD and *n* (%)

#### Procedural data

VT ablation was performed with an exclusively endocardial approach for 73.7% of patients and combined endo- and epicardial approaches for the remaining patients. A multielectrode mapping catheter was used for 17.5% of the patients, and scars were also checked with a contact sensor catheter to avoid false scar detection due to poor contact. The average mapping points were 383.8 ± 201.2 (range 158–873) in the endocardium and 833.8 ± 373.9 (range: 239–1673) in the epicardium. To avoid mixing 3.5-mm catheters and multielectrode mapping catheter, only voltage maps performed with contact force 3.5-mm catheter have been analyzed to delineate CCs in this study. The transeptal approach in LV mapping was used in 56.8% of the patients. Non-inducibility and complete abolition of late potentials were achieved in 70.9% and 71.8% of patients, respectively. Major complications occurred in 6.3% of patients. Procedure characteristics are listed in Table 2.

**Table 2:** Baseline characteristics based on the VT ablation procedure

	Total patients = 57
Endocardial mapping, points	383.87 ± 201. 21
Epicardial mapping, points	833.86 ± 373.99
Procedure duration, min	189. 42 ± 82.89
RF application time, min	14.93 ± 10.53
Fluoroscopy time, min	15.29 ± 8.54
Pentaray	16 (28)
Retroartic access	39 (68)
Epicardial access	6 (10)
Transeptal puncture	18 (32)
Number ventricular tachycardia induced	1.81 ± 1.1
Major procedural complications	5 (9)
Recurrence at 18 months follow-up	21 (34)
Successful procedure	51 (89)

Values are mean ± SD and *n* (%)

#### Conducting channels and voltage thresholds

**Fig. 2:**
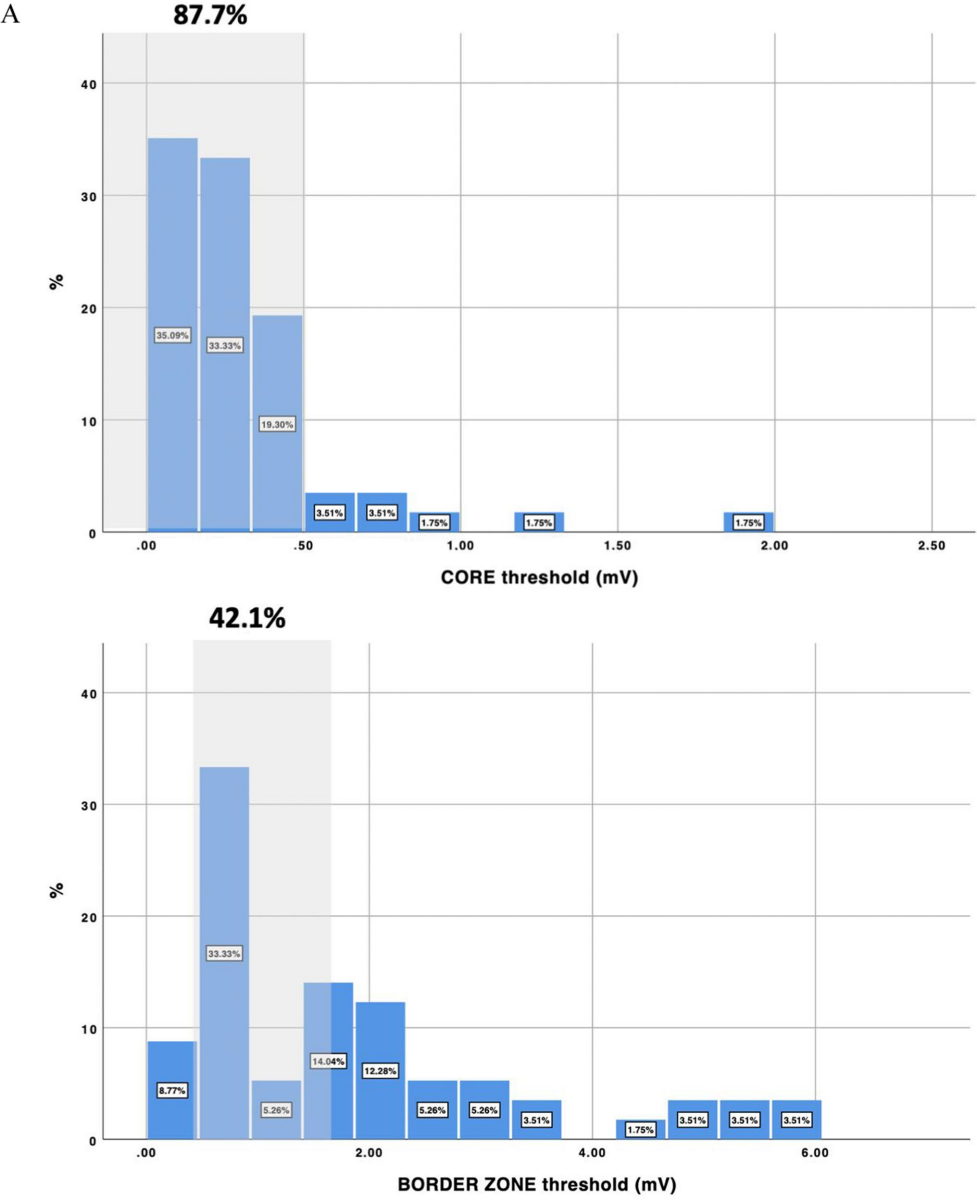

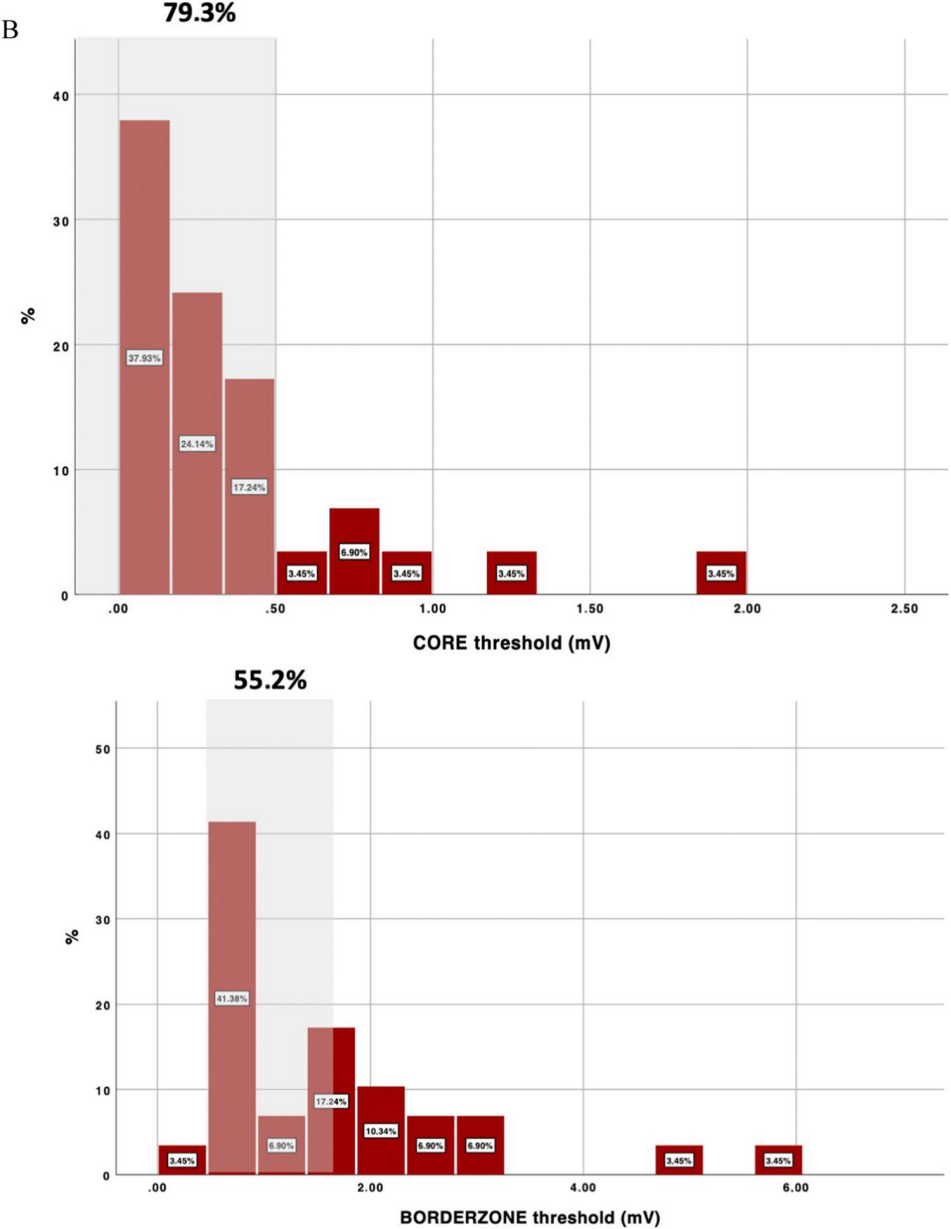

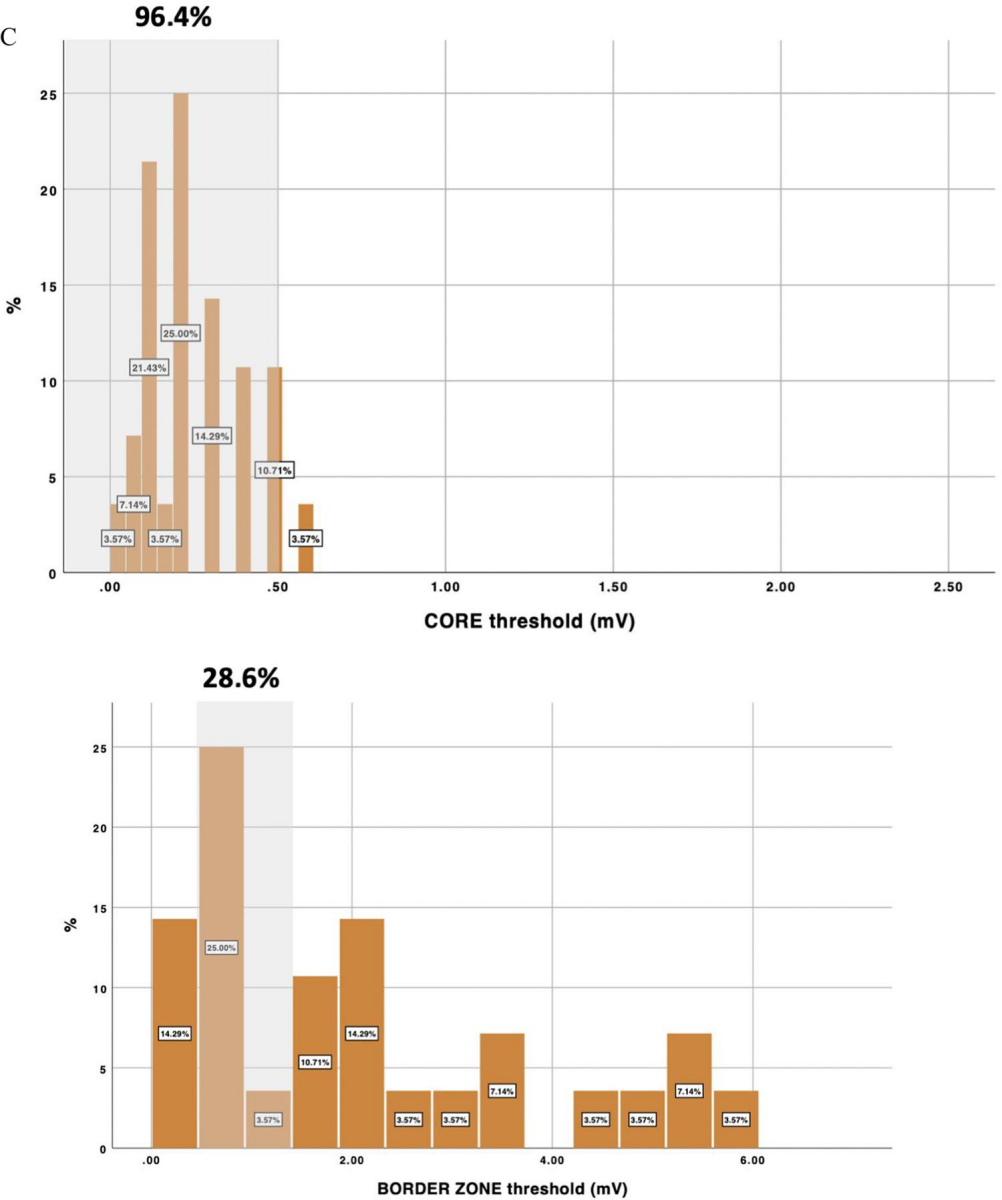
Voltage threshold distribution for the scar and border zone tissue in delineating conduction channels. In panels **A**, **B**, and **C**, the distribution is shown for the overall population (**A**), ischemic patients (**B**), and non-ischemic patients (**C**). The proportion of patients correctly identified using conventional thresholds is shown in gray shadow

**Fig. 3:**
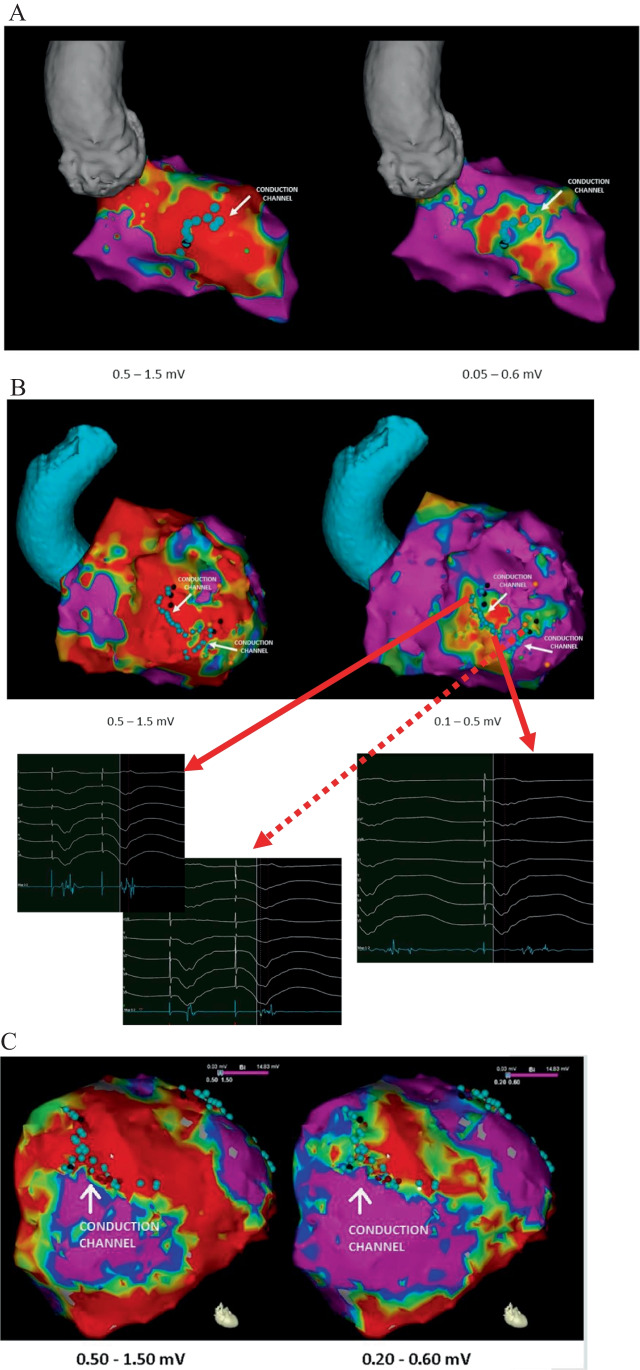
Examples of the poor performance of conventional thresholds (0.5–1.5 mV) (left panel) and the tailored voltage in delineating late potential channels (right panel). Voltage maps are shown with the usual color code: healthy tissue = purple, core scar = red, border zone = yellow, green and blue. Channels with late potentials are marked with blue dots. Thresholds used in every map are listed in the bottom. Patient with ischemic heart disease is shown in panels **A** and **B** with endocardial anterior myocardial infarction and conducting channel in anterior wall in panel **A** and transmural anterior myocardial infarction with epicardial conducting channel in panel **B**. In panel **B**, some examples of electrograms of late potentials in the entrance of the channels (so-called early late potentials: first two examples) and inside the channels (typical late potentials seen after QRS complex: right panel) are shown. In panel **C**, a patient with left ventricular arrhythmogenic cardiomyopathy and epicardial substrate is shown

The mean voltages that better identified the core tissue and border zone for delineating conducting channels were 0.32 (0.02–2 mV) and 1.84 (0.3–6 mV), respectively. These values were different between patients with ICM (0.39–1.61) vs NICM (0.24–2.07) but not to a statistically significant degree (*p* = 0.09). Regarding the currently accepted thresholds, 0.5 mV correctly identified the core in 87.7% of patients, while 1.5 mV correctly identified the border zone only in 42.1% of the patients (Fig. 2). The diagnostic yield of these values was better in ICM patients. In this sense, there was a trend of better diagnostic yield in identifying core tissue in ICM patients with a threshold of 0.5 mV (96.4% in ICM vs 79.3% in non-ICM, *p* = 0.102), and there was a significant difference between the percentages of ICM and non-ICM patients for whom the border zone was identified with a 1.5 mV threshold (55.2% in ICM vs 28.6% in NICM, *p* = 0.042). Any other predictors were found in relation to the diagnostic yield of current accepted thresholds (Table 3). Some examples of the value of conventional thresholds versus tailored thresholds are shown in Fig. 3.

**Table 3:** Diagnostic yield of current voltage thresholds

	CORE			BORDER ZONE		
	Standard	Non-standard	*P* value	Standard	Non-standard	*P* value
Hypertension	71.4%	50%	0.427	58.3%	48.5%	0.462
Diabetes mellitus	24%	14.3%	1	25%	21.2%	0.736
Atrial fibrillation	20%	28.6%	0.630	25%	18.2%	0.533
Arterial access	25%	23.2%	0.963	22.3%	25.4%	0.875
Transeptal access	55.6%	100%	1	80%	40.9%	0.07
**Ischemic heart disease**	**46%**	**87.7%**	**0.07**	**66.7%**	**39.4%**	**0.04**
Number of mapping points*	558.2 ± 43.4	794.3 ± 211.5	0.31	631 ± 79.1	555.3 ± 55.9	0.42

^*^Values are mean ± SD and *n* (%). Values in bold are those with statistical significant differences

### Follow-up

During 32.9 ± 21.2 months (range: 12–47 months), 41.4% of the patients presented VT recurrence (24.3% during first year after ablation). Despite recurrence, there was an overall reduction in VT burden (preprocedural: 3.5 [1.3–13.9] vs postprocedural: 0.001 [0–0.69] median VT episodes per year, *p* = 0.001) and also in patients with VT recurrence with a decrease of 69.2% (median of the VT episodes per year: preprocedural 2.8 [1.1–8.8] vs postprocedural 0.88 [0.5–2.28], *p* < 0.001).

## Discussion

The present study analyzed the performance of conventional voltage thresholds in electroanatomical mapping to identify the channels responsible for ventricular tachycardia determined by EGM characteristics. The main results of this study are the moderate diagnostic yield of conventional thresholds in identifying scars, especially border zone tissue, and the poorer performance in patients with NICM. These findings suggest that voltage mapping is not enough for substrate VT ablation and other methods as EGM characteristics must be considered in these procedures.

Ventricular arrhythmias in patients with structural heart disease are related to re-entry circuits with slow conduction channels in border zone tissue between areas of blocked conduction related to core scar tissue [11]. These regions are also called conducting channels (CCs). These CCs can be accurately identified with the aid of electroanatomical maps (EAMs) obtained during ablation [3, 12]. In these areas, reduced conduction velocity and nonuniform anisotropic conduction produce areas of delayed, fractionated electrical activity that can persist even after inscription of the QRS complex during sinus rhythm [13]. These electrograms can be detected as fractionated EGMs at the end of the QRS or after the end of QRS (late potentials). All of these EGMS are often considered LAVAs (local abnormal ventricular activity) and their elimination has been proposed as an endpoint in VT ablation [14] after several studies demonstrated their relation with VTs [15]. The CCs were first described by voltage mapping as the area of the border zone between areas of unexcitable scar tissue. To identify these areas, a threshold of 1.5 mV for the border zone and 0.5 mV for the core was described 20 years ago [5]. Since that, despite some works suggesting other thresholds (i.e., 1.8 mV), especially for the border zone [6], these values are still recommended in the most recent clinical practice guidelines [4]. To date, the performance of conventional thresholds has been tested only against fibrosis detected by late gadolinium enhancement cardiac magnetic resonance in ICM [16] and NICM [17] patients, and as in our study, when applied to patients with NICM, the correlation between voltage map and CMR was poor.

As reported in many recent studies, voltage amplitude and duration are dependent on many factors, such as tissue contact, wavefront orientation, and interelectrode distance [18]. Following the description of the current thresholds [5], many new developments in mapping and ablation catheters have arisen. One of the key factors in determining voltage amplitude is the contact between the mapping catheter with the tissue. In this sense, in the study in which the current thresholds were first described [5], the catheter used for mapping had no sensor to measure the contact with the tissue. In recent years, contact sensor catheters have been widely used, and the current VT ablation guidelines recommend their use [4]. Some recent studies have demonstrated the relation between contact force and the better definition of scar tissue [19]. In our study, we routinely used a contact force catheter to assure that the low amplitude of voltage was not due to poor catheter-tissue contact.

In addition to using contact force catheters, to our knowledge, this is the first study that associates voltage thresholds with conducting channels defined purely by electrical EGM properties. Our gold standard for defining a conduction channel is the presence of LAVAs and a pattern of activation with late potentials in the middle of the channel. As mentioned before, substrate modification based on late potential abolition (with different techniques) has been shown to have better long-term results. Therefore, the detection of these late potentials plays a key role in VT ablation. In this sense, finding a threshold that better identifies conducting channels could help to improve the VT ablation results.

In our study, the performance of conventional thresholds in defining scar and especially border zone tissue ranged from poor to moderate. This was even more marked in NICM patients. NICM is a more heterogeneous group of diseases, and the distribution of substrate is highly variable in terms of not only the distribution through the myocardium (endocardium, transmural, midmyocardium, epicardium) but also the fibrosis pattern [20]. In this sense, in a small group of NICM patients who underwent necropsy, it was shown than bipolar and unipolar voltage electroanatomical maps had poor agreement with the substrate found in the histological analysis. The heterogeneous distribution of fibrotic tissue can also explain, in our study, the poorer performance of conventional thresholds and the wider range of adequate thresholds to define border zone and scar tissue found in our study in NICM patients.

### Limitations

There are some limitations of our study that must be addressed.

One of the main limitations of the study is the retrospective nature. However as the main endpoint of our study refers to electroanatomical mapping, we consider that the retrospective design has a limited effect in our results. Because our results are not related to follow-up and the main goal of the study is to analyze the performance of current thresholds in relation to EGM-defined conducting channels, the benefits of a prospective design are limited. In addition, all patients were recruited prospectively in the general VT ablation database.

Another very important limitation is that EAM was performed without a high-density mapping catheter. Although it has been demonstrated that the definition of scar tissue is more precise with these catheters due to their lower interelectrode space, there are no available high-density mapping catheters with contact tissue sensor. In this sense, because we wanted to analyze the voltage amplitude, we considered the use of a contact sensor mapping catheter to be a key factor. However, the voltage thresholds could be different from those obtained with this type of catheters.

Some limitations must be commented about EGM characteristics and CCs. Firstly, because the scar dechanneling technique has been used as ablation method in this study (no induction at the beginning of the procedure), in many patients it has not been proved that ablated CCs were linked with a clinical VT. However, several studies have shown that if ablation is limited to areas linked to induced or clinical VT, the recurrence rate is extremely high and that residual late potentials after the ablation, despite the patient is not inducible, are related with a higher recurrence rate [1, 21]. These data strongly suggest that channels not proved to conduct a VT during the ablation procedure can be the conduction channel of another VT during the follow-up. Furthermore, the presence of CCs itself (without validation with clinical VT) is linked with arrhythmogenicity [22]. Finally, despite CCs are clearly defined and are one of the main mechanisms of re-entry VT, some VTs can be related with functional delay so EGM characteristics and therefore CCs depend also on pacing site and pacing cycle length. In addition, CCs can have a 3D architecture so can be missed in the EAM because of intramural paths.

Finally, it must be considered that voltage mapping in our study has been performed during RV pacing. The accepted 0.5–1.5 mV thresholds were described during sinus rhythm and activation wavefront could affect voltage amplitude, especially in septal scars. On the other hand, it is shown that RV pacing can unmask late potentials that, indeed, are the areas that are critical for VT circuits and that are the areas that must be ablated to avoid VT recurrences. Overall, as the goal of the study was to analyze the voltage thresholds to delineate LP channels, activation from RV pacing seems more appropriate than sinus rhythm mapping.

## Conclusions

The conventional 0.5 mV and 1.5 mV voltage thresholds used to define the VT substrate have limited value in delineating conducting channels in terms of late potential activation, especially in patients with NICM. New voltage cutoffs are needed to better analysis of VT substrate specially to define border zone tissue. Indeed, our study confirms that despite voltage scanning can be helpful in some patients, voltage mapping is not sufficient and EGM characteristics analysis may be considered in substrate VT analysis.
